# Biliary stent insertion after stone clearance in patients awaiting cholecystectomy: Systematic review and meta-analysis

**DOI:** 10.1055/a-2586-6007

**Published:** 2025-05-12

**Authors:** Marco Valvano, Daniele Balducci, Antonio Vinci, Andrea Ghezzi, Shirin Djahandideh, Stefano Fabiani, Gianpiero Stefanelli, Silvia Buccilli, Amedeo Montale, Filippo Antonini, Luca Maroni, Chiara Campanale

**Affiliations:** 1 367958 Department of Health Sciences, University of L'Aquila Department of Health Sciences, L'Aquila, Italy; 2 9336 S.C. Gastroenterologia, Ente Ospedaliero Ospedali Galliera, Genova, Italy; 3 9294 Università Politecnica delle Marche, Ancona, Italy; 4 9318 University of Rome Tor Vergata, Roma, Italy; 5 9336 Ente Ospedaliero Ospedali Galliera, Genova, Italy; 6 18637 Pescara Hospital, Pescara, Italy; 7 Gastroenterology and Digestive Endoscopy, Augusto Murri Hospital, Fermo, Italy

**Keywords:** Pancreatobiliary (ERCP/PTCD), Stones, ERC topics, Cholangioscopy

## Abstract

**Background and study aims:**

Laparoscopic cholecystectomy is the standard treatment for patients with cholecystitis or gallbladder stones after common bile duct (CBD) clearance. According to the sequential strategy, cholecystectomy should be performed within 2 weeks after CDB clearance with endoscopic retrograde cholangiopancreatography (ERCP). However, in real-life experience, the average waiting time is 60 to 180 days. We aimed to evaluate the clinical rationale for prophylactic stent placement in CBD to prevent recurrent biliary events.

**Patients and methods:**

This systematic review and meta-analysis was performed following a protocol designed a priori (PROSPERO: CRD42024564804; July 13, 2024). All published studies involving patients who had undergone ERCP for CBDs and who were awaiting cholecystectomy were included.

**Results:**

At the end of the revision process, four full texts including 755 patients were included in the meta-analysis. The odds ratio (OR) for symptom recurrence in patients awaiting cholecystectomy was 0.74 (95% confidence interval [CI] 0.30-1.79; I ^2^ 67%). The pooled OR for adverse event occurrence was 0.74 (95% CI 0.45-1.24) in the stent group. The post-ERCP pancreatitis and cholangitis risk were 0.76 (95% CI 0.25-2.34) and 0.92 (95% CI 0.31-2.67), respectively.

**Conclusions:**

This meta-analysis showed no benefit for stent insertion after bile duct clearance in patients scheduled for delayed cholecystectomy. Further randomized controlled trials with bigger cohorts are needed to assess any benefit for this procedure, which in the meantime, cannot be recommended.

## Introduction

Gallstones are a common issue in developed countries with a prevalence of 10% to 15% and an overall cumulative incidence formation of 0.60% per year 1 .

Common bile duct stones (CBDSs), if detected, should be removed, also in asymptomatic patients, for their risk of complications such as pancreatitis, cholangitis, and obstruction of the bile duct 2 . Complications of CBDSs are often the initial onset of a patient affected by gallstones disease and this is why endoscopic retrograde cholangiopancreatography (ERCP) is usually performed before cholecystectomy.

According to current guidelines, laparoscopic cholecystectomy is the standard treatment for patients with CBD and gallbladder stones after CBD clearance, being associated with a lower mortality risk 3 . According to the sequential strategy, the cholecystectomy should be performed within 2 weeks from ERCP to reduce recurrence of biliary events or complications. The clinician could also consider intraoperative ERCP with the rendezvous technique in patients with CBDSs undergoing cholecystectomy in order to perform two procedures simultaneously in the same session 3 .

In real-life experience, the average waiting time for cholecystectomy after ERCP is 60 to 180 days 4 5 . The main reasons for deferred cholecystectomy include unstable patient medical status and overall limited availability of operative schedules, especially for long waiting lists; the COVID-19 period influenced the data for the surgical lockdown 6 7 . A comparative study showed that early laparoscopic cholecystectomy is the cheapest treatment, considering the costs of health care in patients that wait too long for surgery and develop biliary events 8 .

Data from a retrospective study demonstrated that in patients with deferred cholecystectomy after CBD clearance, recurrent biliary events occurred in 28.5% of the population at a median time of 34 days. Cholecystitis was the most frequent recurrent biliary event (47%), with the first episode occurring at a median time of 29.5 days 9 .

Given this difference between guidelines and real-life practice, the prophylactic placement of a transpapillary stent in the CBD or in the gallbladder through cystic duct cannulation has been proposed during ERCP, after CBD clearance, with the purpose of preventing recurrence of biliary events (i.e., pancreatitis, cholangitis, cholecystitis, bile duct obstruction). However, data from the literature regarding this prophylactic stent placement is scanty and controversial: indeed, conclusions from studies in the literature differ from each other 10 11 12 13 .

The objective of this meta-analysis was to evaluate the clinical rationale for prophylactic stent placement in CBD to prevent recurrent biliary events.

## Patients and methods

### Protocol and registration

This systematic review and meta-analysis was performed following a protocol designed a priori (PROSPERO n: CRD42024564804; 13 July 2024) and reported according to the Preferred Reporting Items for Systematic Reviews and Meta-Analyses guidelines (PRISMA) guidelines ( **Supplementary Table 1** ) 14 .

### Search and selection process

An electronic search for relevant publications (without language restriction or date of publication restriction) was performed by two investigators (A.G. and S.S.). Studies were identified using the following database: PubMed/MEDLINE, Scopus, and CENTRAL (data of last search 16/07/2024; **Supplementary Table 2** ).

Each of the relevant publications (previous review articles and included studies) reference sections and Google Scholar were also screened for other applicable publications.

ClinicalTrials.gov was searched for unpublished completed trials. Relevant abstracts from United European Gastroenterology week conference and Digestive Disease Week were also screened.

### Eligibility criteria and data items

Studies of patients who underwent ERCP for CBD obstructive symptoms (e.g. CBDs; gallstone pancreatitis) with complete biliary clearance and awaiting cholecystectomy (performed at least after 1 month) were included. Inclusion criteria for studies were: 1) patients aged 18 years and older undergoing ERCP; 2) randomized study of interventions (RCTs) or non-randomized studies of interventions (NRSIs) including patients with stent positioning compared to no stent positioning group after CBD clearance; 3) complete biliary clearance at ERCP; 4) patients waiting for cholecystectomy (performed after at least 1 month). Exclusion criteria were patients with biliary malignant obstruction and indwelling prothesis.

### Outcome of interest

The primary outcome of this meta-analysis was a composite dichotomous variable, including signs of clinical recurrence evaluated as: 1) need for a new/emergency ERCP; 3) recurrence of obstructive symptoms; 4) gallstone pancreatitis; and 5) cholecystitis.

As a secondary objective, we planned to assess risk of post-ERCP adverse events (AEs). In particular, data concerning post-ERCP pancreatitis (PEP), cholangitis, and post-ERCP bleeding were collected: 1) post ERCP AEs (PEP, cholangitis, post procedure bleeding); 2) given data availability, subgroup analysis, or sensitivity analysis involved; 3) type of stent used; and 4) type of AEs.

### Data extraction (selection and coding)

Two reviewers (A.G. and S.S.) independently reviewed the literature according to predefined protocol; any disagreement was resolved by consensus or by recourse to a third author (V.M.).

Each reviewer extracted the following data variables in a pre-made template: title and reference details (first author, journal, year, country), study population characteristics (number of patients, gender, age, study design), outcome data (recurrence of choledocholithiasis/cholecystitis, gallstone pancreatitis, postoperative biliary leak, need for repeat ERCP during the waiting period, and incidence of post ERCP AEs).

All data were recorded independently by both literature reviewers in separate databases and were compared at the end of the reviewing process to limit selection bias. Any disagreement was resolved by consensus or by recourse to a third author (V.M.).

Authors of eligible studies were contacted for additional information about the occurrence of the inconsistency of reported results during data extraction, or in case of lacking\missing data.

### Risk of bias (quality) assessment

Quality of included studies was assessed by two reviewers (V.M. and D.B.) using the Risk of Bias tool (RoB 2.0) for RCTs and ROBINS-I for the NRSI 15 16 . To assess the quality of evidence, we used the GRADE (Grading of recommendations, Assessment, Development, and Evaluation) approach, which classifies evidence as high, moderate, low, or very low quality based on consideration of risk of bias, consistency, directness, precision, and publication bias 17 18 19 .

### Strategy for data synthesis

Primary outcome was expressed as pooled odds ratios (ORs) with 95% confidence intervals (CIs). The OR of each individual study was pooled using a random-effects model. Heterogeneity was investigated using the chi² test and I² statistic. A 95% prediction interval was calculated assuming normal distribution of the effect. For NRSI, mediational E-values (for ORs of 2 and 0.5) were calculated, to estimate how strong an unmeasured confounder should be to move the observed OR to such target values 20 21 22 .

We planned to perform publication bias investigation using funnel plots and Egger’s regression test in case of outcomes with 10 or more available studies 23 .

Stata v.17.1 and Review Manager (RevMan) v.5.4 statistical software was used for analysis, with the statistical significance threshold set at 0.05.

### Sensitivity analysis

A sensitivity analysis was scheduled after excluding studies without a published full-text and/or moderate/critical risk of bias (evaluated with the RoB 2.0 and the ROBINS-I). Also, NRSI vs RCTs subgroup analysis was planned. To investigate for power issues in published literature, trial sequential analysis (TSA) was performed, as suggested by Thorlund et al 24 .

TSA was performed using the power calculation data provided by Sasani et al. 11 , considering a recurrence of choledocholithiasis after clearance on ERCP in approximately 30% of patients, and expecting a reduction due to intervention to 5% of patients 25 26 . Thus, estimating that 30% of patients in the no stent group will have recurrence, and by placing a stent, this would be reduced to 5%.

### Summary of findings and GRADE profile

The main findings of this meta-analysis concerning the certainty of the evidence, and the magnitude of the effect of the interventions examined, are presented in Table 1 , according to GRADE recommendations.

**Table:** **Table 1** Summary of findings.

Certainty assessment							Summary of findings		
Participants (studies) Follow-up	Risk of bias	Inconsistency	Indirectness	Imprecision	Publication bias	Overall certainty of evidence	Study event rates (%)		Relative effect (95% CI)
							With stenting	With no stenting	
**Stent vs no stent**									
755 (2 RCTs; 2 NRSIs)	Not serious	Very serious ^*^	Not serious	Serious ^†^	None	⊕○○○ Very low*,†	53/429 (12.3%)	55/326 (16.8%)	**OR 0.74** (0.30 to 1.79)
**Adverse events**									
593 (2 RCTs; 1 NRSI)	Not serious	Serious‡	Not serious	Not serious	None	⊕⊕⊕⊕ High	32/902 (3.5%)	35/767 (4.5%)	**OR 0.74** (0.45 to 1.24)
CI, confidence interval; NRSI, non-randomized study of intervention; OR, odds ratio; RCT, randomized clinical trial. *High heterogeneity only in part due to different study design. ^†^ Wide confidence interval due to different population characteristics and intervention types ^‡^ Non-negligible heterogeneity despite the subgroup analysis									

## Results

**Fig. 1:**
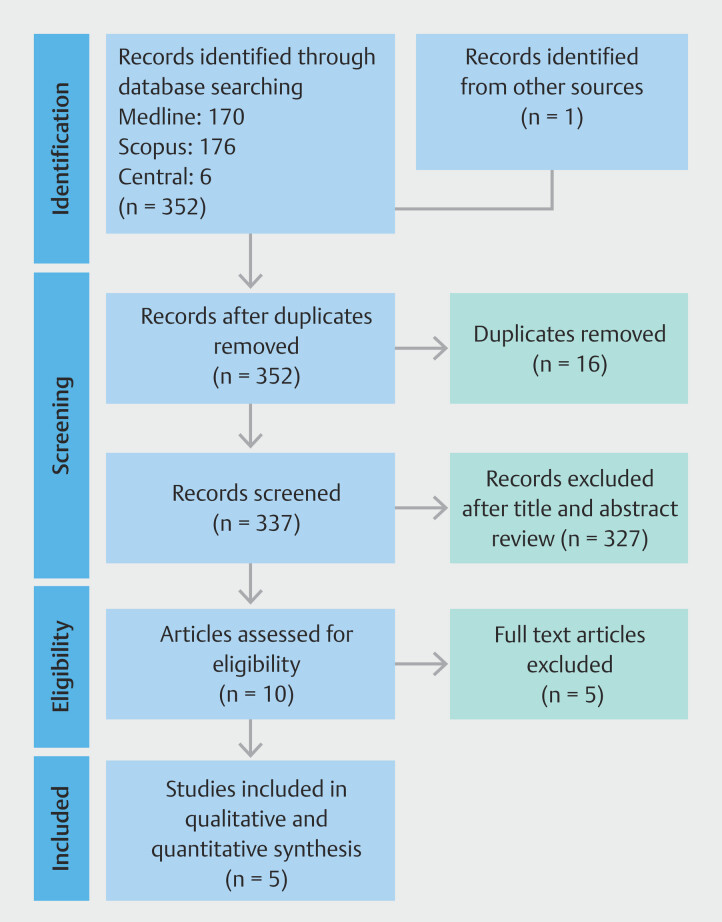
PRISMA flow-diagram 27 .

The results of the literature review are summarized in Fig. 1 . The initial search identified 352 articles, from which 17 duplicate records were excluded. Following a comprehensive screening and revision process, four full-text articles, encompassing data from 755 patients, were included in the meta-analysis ( Fig. 1 ). The included studies comprised two RCTs and two NRSI.

Characteristics of the four selected studies are reported in Table 2 . The RCTs and the NRSI included in our meta-analysis had an intervention group treated with plastic CBD stents (two pigtail, one standard stent, and one not specified). Risk of bias of the included studies assessed by the RoB 2.0 and ROBINS-I is summarized in **Supplementary Fig. 1** and **Supplementary Fig. 2** .

**Table:** **Table 2** Baseline characteristics of included studies.

Study	Design	Country	Case group (stenting)	Control group (no stenting)	Male, n (%)	Age ^*^ , case	Type of stent	Timing of LC	Primary outcome
Hormati 2022	RCT	Iran	20	20	11 (55)	Case: 51.9 ± 13.06 Control: 51.8 ± 13.59	Standard straight (10F)	Case: 4 weeks Control: 4 weeks	Recidive CBDs
Sasani 2023	RCT	India	35	35	12 (35,3)	Case: 46 (34–59.75) Control: 46 (30.25–61.75)	Double pigtail (7- 10F)	Case: 12 weeks Control: 12 weeks	Recidive CBDs
Verzhbitsky 2013	Retrospective	Israel	110	52	Case: 59 (53,7) Control: 17 (33,3)	Case: 61.4 ± 17.7 Control: 58.3 ± 19.4	NA	Case: 7 weeks (mean) Control 6 weeks (mean)	Biliary event after stent insertion
Choi 2021	Retrospective	Korea	264	219 ^†^	Case: 153 (58.0) Control: 121 (55,3)	Case: 63.8 ± 15.3 Control: 61.4 ± 17.2	Double pigtail (7F)	NA	Recidive CBDs
*Patients with severe cholangitis, ≥ 3 stones, periampullary diverticulum were candidates for stent positioning. ^†^ Mean ± SD or median (interquartile range). CBD, common bile duct; LC, laparoscopic cholecystectomy; NA, not applicable; RCT, randomized controlled trial.									

Table 1 shows the main results of the review concerning the certainty of the evidence and magnitude of the effect of the interventions examined. Characteristics of excluded studies are summarized in **Supplementary Table 3** in the appendix.

### Efficacy of prophylactic stent insertion

**Fig. 2:**
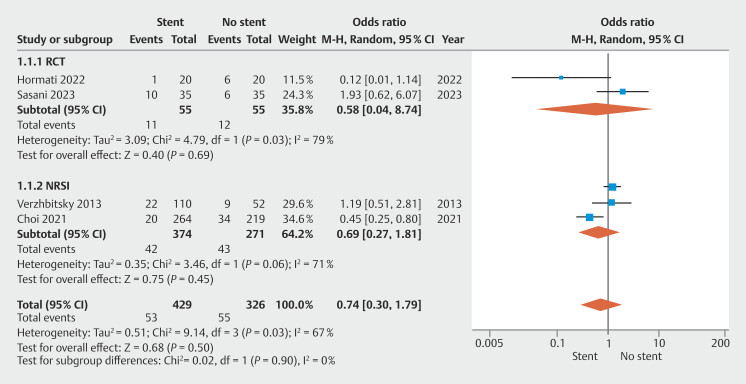
Clinical recurrence in stent vs no stent group.

Among the four included studies, the pooled OR for a recurrence of symptoms in patients waiting for cholecystectomy after stent insertions was 0.74 (95% CI 0.30–1.79; I ^2^ 67%) ( Fig. 2 ). In subgroup analysis, the ORs were 0.58 (95% CI 0.04–8.74) and 0.69 (95% CI 0.37–1.81) among RCTs and NRSIs, respectively ( Fig. 2 ).

### Post-ERCP adverse events

Among the included studies, three reported data concerning both cholangitis and PEP 11 13 28 and one reported data on post-ERCP bleeding 13 .

**Fig. 3:**
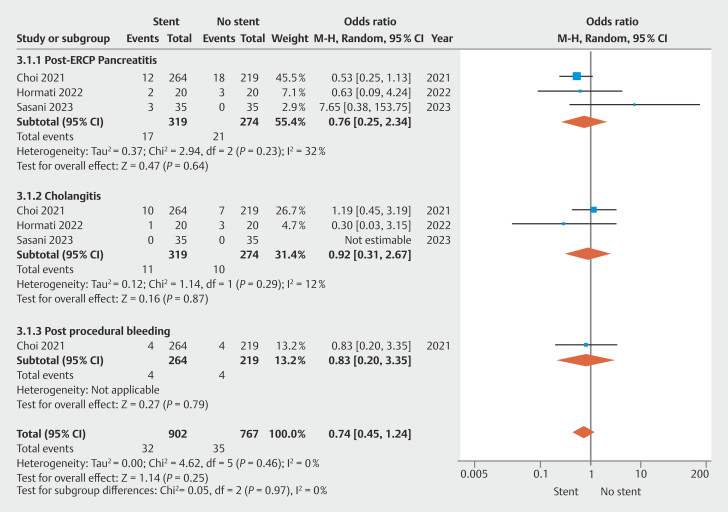
Risk of post-ERCP adverse events.

The pooled OR for AE occurrence was 0.74 (95% CI 0.45–1.24) in the stent group. The PEP and cholangitis risk were 0.76 (95% CI 0.25–2.34) and 0.92 (95% CI 0.31–2.67), respectively ( Fig. 3 ).

### Sensitivity analyses and investigation of publication bias

To investigate robustness of the results, a subgroup analysis including only studies with double pigtail stent used in the intervention group was conducted. The OR was 0.85 (95% CI 0.20–3.55) with a substantial high heterogeneity (I ^2^ 80%) ( **Supplementary Fig. 3** ).

Publication bias could not be adequately assessed using the funnel plot or Egger’s regression test due to the small number of selected studies.

Data concerning the risk of bias for each included study are reported in **Supplementary Fig. 1** and **Supplementary Fig. 2** in the appendix.

All the included RCTs presented a low risk of bias in all the domains 11 28 . Also the two NRSIs presented a low risk of bias at the ROBINS-I evaluation 13 29 . Because NRSIs are inherently more prone to bias due to confounding than RCTs, and because it was not possible to adjust by internal stratification, mediational E-values were calculated. We found that to completely explain away the observed effect size moving actual OR to 2, an unmeasured confounder associated with both ERCP and AEs with approximate risk ratios of 3.18-fold, above and beyond the measured covariates, could suffice, but weaker confounding could not. To shift the OR to 0.5, an unmeasured confounder associated with both ERCP and AEs with approximate risk ratios of 3.66-fold each, above and beyond the measured covariates, could suffice, but weaker confounding could not.

We performed a sensitivity analysis after exclusion of only one study (Choi 2021) that did not state in its methods sections if the included patients presented with residual gallbladder stones. The pooled OR after its exclusion was 0.94; 95% CI 0.30–2.95 ( **Supplementary Fig. 4** ).

TSA shows that in the current literature, there is something missing in terms of RCTs; keeping analysis power at 80% and alpha error at 5%, considering a recurrence of choledocholithiasis after ERCP clearance in approximately 30% of patients, and expecting a reduction due to intervention to 5% of patients, the cumulative number of patients within an RCT should be 520 patients (260 for each group). However, after the inclusion of NRSIs in TSA, the significance boundary was lowered to 304 patients, and this meta-analysis fit well within this limit, having included 755 patients ( **Supplementary Fig. 5** ).

## Discussion

Our systematic review and meta-analysis, encompassing four studies with a total of 755 patients, sought to evaluate the efficacy of prophylactic biliary stent insertion in patients awaiting cholecystectomy following stone clearance during ERCP. The findings from the meta-analysis indicate no significant difference in clinical recurrence of choledocholithiasis or biliary complications between patients who received biliary stenting after biliary clearance and those who did not.

Gallstone disease is highly prevalent worldwide, and patients with choledocholithiasis are at risk of developing serious complications such as gallstone pancreatitis and cholangitis. For patients presenting with both cholecystitis and choledocholithiasis, treatment must address both biliary obstruction and cholecystectomy 30 .

Delaying cholecystectomy has been shown to substantially increase risk of recurrent biliary events, including pancreatitis, acute cholecystitis, cholangitis, and biliary colic, as compared with early cholecystectomy. A Cochrane systematic review highlighted complications faced by patients during the waiting period for delayed surgery, which averaged 4.2 months 31 . These complications included severe acute pancreatitis, empyema, gallbladder perforation, acute cholecystitis, cholangitis, obstructive jaundice, and recurrent biliary colic requiring hospital visits. Similarly, Lo et al. reported a higher incidence of recurrent gallstone-related events and emergency surgery in the delayed group 32 . Reinders et al. showed that during the waiting period for delayed surgery, 36.2% of patients developed recurrent biliary events, compared with only one patient in the early surgery group 33 . A RCT by Jee et al. also found a significantly higher rate of recurrent biliary events in the delayed group (44.12%) compared with the early group (0%) 31 .

Biliary stenting is crucial in managing choledocholithiasis, particularly when immediate stone removal is not feasible. In fact, it can facilitate stone size reduction and clearance on subsequent procedures and manage biliary obstruction effectively 34 35 . The exact mechanism of action of stenting in this setting remains uncertain; however, it is hypothesized that friction between gallstones and stents during respiratory movements, along with the role of the stent in maintaining continuous bile flow and preventing stone accumulation, contributes to its efficacy 36 .

Prophylactic stent placement in patients scheduled for delayed cholecystectomy aims to maintain continuous biliary drainage and potentially prevent clinical complications arising from gallstone migration from the gallbladder into the bile duct 37 . The potential mechanisms are fragmentation of smaller stones into sludge, which is then expelled through the papilla and spontaneous passage of stones alongside the bile duct stent 38 . However, the literature on effectiveness of prophylactic stenting is mixed, with some studies suggesting a benefit 13 28 39 and others not supporting its use 11 29 40 . The effect of biliary stenting on cholecystectomy outcomes have also been explored. A study by Nair et al. suggested that placing a bile duct stent for 6 weeks before elective laparoscopic cholecystectomy influenced factors such as operative time, conversion rate to open surgery, bile leak rate, and length of hospital stay 41 . Also, Manojkumar et al. showed a significant increase in rates of conversion to open surgery in patients with inserted prophylactic stent 41 . On the other hand, other studies found no surgical AEs associated with biliary stenting and indicated that stent insertion did not predict conversion to open cholecystectomy or prolonged operative time 29 41 42 .

To our knowledge, this is the first meta-analysis aiming to synthesize all available data on this topic. Our results indicate that prophylactic stent placement does not confer a significant benefit in preventing biliary complications in patients awaiting cholecystectomy ( *P* = 0.50). This conclusion remains valid even when the analysis is restricted to studies using double pigtail stents ( *P* = 0.83). Furthermore, given the invasive nature of the procedure, its potential for AEs must also be considered. Our analysis revealed no differences in risk of AEs between the groups ( *P* = 0.25): in particular, no differences were found for PEP ( *P* = 0.64), cholangitis ( *P* = 0.87) or post-procedure bleeding ( *P* = 0.79).

Our findings align with the largest population-based study to date (10.1016/j.igie.2024.04.007), which demonstrated that prophylactic biliary stenting in conjunction with sphincterotomy provided no additional benefit in preventing recurrent biliary events. However, the study reported significantly higher 30-, 60-, and 90-day readmission rates in patients who underwent prophylactic biliary stent placement, highlighting the potential adverse effects of this intervention. However, it is important to note, that the authors were unable to adequately assess bile duct clearance in both patient cohorts, a factor that can significantly influence readmission rates and AEs. Consequently, this study was excluded from our meta-analysis

Even in the absence of significant differences in AEs between the two groups, use of a prophylactic biliary stent raises concerns regarding its potential impact on surgical outcomes. As previously discussed, the relationship between biliary stenting and cholecystectomy outcomes remains a subject of debate. This variability may partially be attributed to differences in timing of cholecystectomy. Notably, a biliary stent, as a foreign body, can promote chronic inflammation and bacterial colonization as early as 1e month post-placement 43 . Furthermore, retrospective data indicate that delaying cholecystectomy beyond 6 weeks constitutes an independent risk factor for postoperative complications 44 . Therefore, early laparoscopic cholecystectomy should be strongly considered to mitigate postoperative complications, particularly in cases where a prophylactic biliary stent has been placed.

This study has some limitations. First, substantial heterogeneity was found across all the outcomes. We attempted to mitigate this through a sensitivity analysis. Specifically, we performed the analysis for the main outcome by considering only studies with a certain type of stent, which could be a confounding factor, and by excluding Choi et al. (2021), the only included study that does not give information concerning residual gallbladder stones. Both analyses resulted in lower but still significant heterogeneity, with confirmation of the previous result. It is important to emphasize that statistical heterogeneity, which may arise from the small number of included studies, does not necessarily indicate clinical heterogeneity. In fact, we can hypothesize that clinical heterogeneity is unlikely, given that all the included studies yielded similarly inconclusive results. Conversely, the TSA revealed a suboptimal sample size when considering only the high-quality data derived from RCTs. However, after including NRSIs in the TSA, the sample size was adequate. However, we cannot exclude the possibility that the suboptimal sample size may have resulted in underpowered findings. Additional data from new studies could help strengthen the conclusions of our meta-analysis. Nevertheless, our results appear robust because numerous sensitivity analyses did not alter the direction of the findings.

## Conclusions

In conclusion, this meta-analysis shows no benefits of stent insertion after bile duct clearance in patients scheduled for delayed cholecystectomy. Further RCTs with bigger cohorts are needed to assess any benefit of this procedure, which, in the meantime, cannot be recommended.

## Supplementary Material

Supplementary Material Zusatzmaterial
