# Supplementary material for: Biliary stent insertion after stone clearance in patients awaiting cholecystectomy: Systematic review and meta-analysis
Source: Endosc Int Open. 2025 May 12;13:a25866007. doi: 10.1055/a-2586-6007 (PMC12080521; doi:10.1055/a-2586-6007)

Supplementary Table 1 PRISMA checklist.

Section and topic	Item #	Checklist item	Location where item is reported
**TITLE**			
Title	1	Identify the report as a systematic review.	**Title page**
**ABSTRACT**			
Abstract	2	See the PRISMA 2020 for Abstracts checklist.	**Abstract**
**INTRODUCTION**			
Rationale	3	Describe the rationale for the review in the context of existing knowledge.	**Introduction**
Objectives	4	Provide an explicit statement of the objective(s) or question(s) the review addresses.	**Introduction**
**METHODS**			
Eligibility criteria	5	Specify the inclusion and exclusion criteria for the review and how studies were grouped for the syntheses.	**Methods**:<br>Eligibility criteria and data items:<br>- Inclusion criteria<br>- Exclusion criteria
Information sources	6	Specify all databases, registers, websites, organizations, reference lists and other sources searched or consulted to identify studies. Specify the date when each source was last searched or consulted.	**Methods**:<br>- Search and selection process
Search strategy	7	Present the full search strategies for all databases, registers and websites, including any filters and limits used.	**Supplementary table and figure**:<br>- Supplementary Table 1
Selection process	8	Specify the methods used to decide whether a study met the inclusion criteria of the	**Methods**:<br>- Eligibility criteria and data item

Section and topic	Item #	Checklist item	Location where item is reported
		review, including how many reviewers screened each record and each report retrieved, whether they worked independently, and if applicable, details of automation tools used in the process.	
Data collection process	9	Specify the methods used to collect data from reports, including how many reviewers collected data from each report, whether they worked independently, any processes for obtaining or confirming data from study investigators, and if applicable, details of automation tools used in the process.	**Methods**:<br>- Data extraction
Data items	10a	List and define all outcomes for which data were sought. Specify whether all results that were compatible with each outcome domain in each study were sought (e.g. for all measures, time points, analyses), and if not, the methods used to decide which results to collect.	**Methods**:<br>- Data extraction<br>- Strategy for data synthesis
	10b	List and define all other variables for which data were sought (e.g. participant and intervention characteristics, funding	**Methods**:<br>- Analysis of subgroups or subsets<br>- Strategy for data synthesis

Section and topic	Item #	Checklist item	Location where item is reported
		sources). Describe any assumptions made about any missing or unclear information.	
Study risk of bias assessment	11	Specify the methods used to assess risk of bias in the included studies, including details of the tool(s) used, how many reviewers assessed each study and whether they worked independently, and if applicable, details of automation tools used in the process.	**Methods:**<br>- Risk of bias (quality) assessment
Effect measures	12	Specify for each outcome the effect measure(s) (e.g. risk ratio, mean difference) used in the synthesis or presentation of results.	**Methods:**<br>- Strategy for data synthesis
Synthesis methods	13a	Describe the processes used to decide which studies were eligible for each synthesis (e.g. tabulating the study intervention characteristics and comparing against the planned groups for each synthesis (item #5)).	**Methods:**<br>Eligibility criteria and data items:<br>- Inclusion criteria<br>- Exclusion criteria
	13b	Describe any methods required to prepare the data for presentation or synthesis, such as handling of missing summary statistics, or data conversions.	**Methods:**<br>- Analysis of subgroups or subsets<br>- Strategy for data synthesis
	13c	Describe any methods	**Methods:**

Section and topic	Item #	Checklist item	Location where item is reported
		used to tabulate or visually display results of individual studies and syntheses.	- Summary of findings and GRADE profile
	13d	Describe any methods used to synthesize results and provide a rationale for the choice(s). If meta-analysis was performed, describe the model(s), method(s) to identify the presence and extent of statistical heterogeneity, and software package(s) used.	**Methods:**<br>- Analysis of subgroups or subsets<br>- Strategy for data synthesis
	13e	Describe any methods used to explore possible causes of heterogeneity among study results (e.g. subgroup analysis, meta-regression).	**Methods:**<br>- Analysis of subgroups or subsets<br>- Strategy for data synthesis
	13f	Describe any sensitivity analyses conducted to assess robustness of the synthesized results.	**Methods:**<br>- Analysis of subgroups or subsets<br>- Strategy for data synthesis
Reporting bias assessment	14	Describe any methods used to assess risk of bias due to missing results in a synthesis (arising from reporting biases).	**Methods**:<br>- Risk of bias (quality) assessment
Certainty assessment	15	Describe any methods used to assess certainty (or confidence) in the body of evidence for an outcome.	**Methods:**<br>- Summary of findings and GRADE profile
**RESULTS**			
Study	16a	Describe the results of	**Results**

Section and topic	Item #	Checklist item	Location where item is reported
selection		the search and selection process, from the number of records identified in the search to the number of studies included in the review, ideally using a flow diagram.	**Figure 1:** PRISMA flow diagram
	16b	Cite studies that might appear to meet the inclusion criteria, but which were excluded, and explain why they were excluded.	**Supplementary table and figure**:<br>- Supplementary table 2
Study characteristics	17	Cite each included study and present its characteristics.	**Results**<br>**Table 1**
Risk of bias in studies	18	Present assessments of risk of bias for each included study.	**Results:**<br>- Sensitivity analyses and investigation of publication bias<br>- **Supplementary table and figure**:<br>- Supplementary Fig. 1 and Supplementary Fig. 2
Results of individual studies	19	For all outcomes, present, for each study: (a) summary statistics for each group (where appropriate) and (b) an effect estimate and its precision (e.g. confidence/credible interval), ideally using structured tables or plots.	**Results**:<br>**Synthesis of results**<br>Efficacy of prophylactic stent insertion<br>Post-ERCP adverse events
Results of syntheses	20a	For each synthesis, briefly summarise the characteristics and risk of bias among contributing studies.	**Results:**<br>- Sensitivity analyses and investigation of publication bias<br>**Supplementary table and figure**:<br>- Supplementary Fig. 1 and Supplementary Fig. 2
	20b	Present results of all	**Results:**

Section and topic	Item #	Checklist item	Location where item is reported
		statistical syntheses conducted. If meta-analysis was done, present for each the summary estimate and its precision (e.g. confidence/credible interval) and measures of statistical heterogeneity. If comparing groups, describe the direction of the effect.	- Sensitivity analyses and investigation of publication bias
	20c	Present results of all investigations of possible causes of heterogeneity among study results.	**Results:**<br>- Sensitivity analyses and investigation of publication bias<br>- Supplementary Fig. 3 and Supplementary Fig. 4
	20d	Present results of all sensitivity analyses conducted to assess the robustness of the synthesized results.	**Results:**<br>- Sensitivity analyses and investigation of publication bias<br>- Supplementary Fig. 3 and Supplementary Fig. 4
Reporting biases	21	Present assessments of risk of bias due to missing results (arising from reporting biases) for each synthesis assessed.	**Results:**<br>- Sensitivity analyses and investigation of publication bias
Certainty of evidence	22	Present assessments of certainty (or confidence) in the body of evidence for each outcome assessed.	Table 2
**DISCUSSION**			
Discussion	23a	Provide a general interpretation of the results in the context of other evidence.	**Discussion**
	23b	Discuss any limitations of the evidence included in the review.	**Discussion**
	23c	Discuss any limitations	**Discussion**

Section and topic	Item #	Checklist item	Location where item is reported
		of the review processes used.	
	23d	Discuss implications of the results for practice, policy, and future research.	**Discussion**
**OTHER INFORMATION**			
Registration and protocol	24a	Provide registration information for the review, including register name and registration number, or state that the review was not registered.	**Methods**:<br>- Protocol and registration (PROSPERO n: CRD42024564804; July 2024)
	24b	Indicate where the review protocol can be accessed, or state that a protocol was not prepared.	**Methods**:<br>- Protocol and registration https://www.crd.york.ac.uk/PROSPERO/#recordDetails
	24c	Describe and explain any amendments to information provided at registration or in the protocol.	**Not performed**
Support	25	Describe sources of financial or non-financial support for the review, and the role of the funders or sponsors in the review.	**Title page - Conflict of interest**
Competing interests	26	Declare any competing interests of review authors.	**Title page**
Availability of data, code and other materials	27	Report which of the following are publicly available and where they can be found: template data collection forms; data extracted from included studies; data used for all analyses; analytic code; any	**Available on request by contacting the corresponding author.**

Section and topic	Item #	Checklist item	Location where item is reported
		other materials used in the review.	

Supplementary Table 2 Research strategy.

**PubMed:** 170 results	(("cholecystitis, acute"[MeSH Terms] OR "cholecystitis"[MeSH Terms] OR "gallstones"[MeSH Terms] OR "choledocholithiasis"[MeSH Terms] OR "cholelithiasis"[MeSH Terms] OR "gallstones"[MeSH Terms] OR "gallstones"[MeSH Terms]) AND ("Delayed laparoscopic cholecystectomy"[All Fields] OR "Delayed cholecystectomy"[All Fields] OR "Awaiting Cholecystectomy"[All Fields] OR (("defer"[All Fields] OR "deferment"[All Fields] OR "deferments"[All Fields] OR "deferred"[All Fields] OR "deferring"[All Fields] OR "defers"[All Fields]) AND ("cholecystectomy"[MeSH Terms] OR "cholecystectomy"[All Fields] OR "cholecystectomies"[All Fields])) OR "late cholecystectomy"[All Fields] OR ("overdue"[All Fields] AND ("cholecystectomy"[MeSH Terms] OR "cholecystectomy"[All Fields] OR "cholecystectomies"[All Fields])) OR ("cholecystectomy"[MeSH Terms] OR "cholecystectomy"[All Fields] OR "cholecystectomies"[All Fields]))) AND **(**("cholangiopancreatography, endoscopic retrograde"[MeSH Terms] OR "cholangiopancreatography, endoscopic retrograde"[MeSH Terms] OR "cholangiopancreatography, endoscopic retrograde"[MeSH Terms] OR "sphincterotomy, endoscopic"[MeSH Terms]) AND ("stents"[MeSH Terms] OR "plastic stent"[All Fields] OR "metal stent"[All Fields] OR "pigtail stent"[All Fields] OR "endoscopic stenting"[All Fields] OR "double pigtail"[All Fields] OR "endoscopic stent"[All Fields] OR "biliary stent"[All Fields] OR "biliary stenting"[All Fields]))) OR ("gallbladder stent")) OR ("gallbladder stenting"))
**Scopus:** 176 results	(cholecystitis OR acute cholecystitis OR gallstones OR choledocholithiasis OR "common bile duct stone" OR "CBDs") AND (cholecystectomy) AND ("Delayed laparoscopic cholecystectomy" OR "Delayed cholecystectomy" OR "Awaiting Cholecystectomy" OR "overdue Cholecystectomy" OR "deferred

	Cholecystectomy" OR "late Cholecystectomy") AND (ERCP OR "cholangiopancreatography, endoscopic retrograde" OR "endoscopic sphincterotomy" OR "sphincterotomy") AND (stent OR "endoscopic stent" OR "plastic stent" OR "metallic stent" OR "biliary stent" OR "pigtail stent" OR "double pigtail stent" OR "gallbladder stent" OR "gallbladder stenting")
**CENTRAL:** 6 results	(cholecystitis OR acute cholecystitis OR gallstones OR choledocholithiasis OR "common bile duct stone" OR "CBDs") AND (cholecystectomy) AND ("Delayed laparoscopic cholecystectomy" OR "Delayed cholecystectomy" OR "Awaiting Cholecystectomy" OR "overdue Cholecystectomy" OR "deferred Cholecystectomy" OR "late Cholecystectomy") AND (ERCP OR "cholangiopancreatography, endoscopic retrograde" OR "endoscopic sphincterotomy" OR "sphincterotomy") AND (stent OR "endoscopic stent" OR "plastic stent" OR "metallic stent" OR "biliary stent" OR "pigtail stent" OR "double pigtail stent" OR "gallbladder stent" OR "gallbladder stenting")

Supplementary Table 3 Full text excluded with reason.

N	Author (year)	Reason for exclusion
1	Manojkumar 2009	Outcome of interest not measured
2	Kobayashi 2024	Gallbladder stenting; outcome of interest not measured
3	Ridtitid 2024	Gallbladder stenting
4	Kawabata 2019	Lack of control group; outcome of interest not measured
5	Cinar 2017	Outcome of interest not measured
6	Chandan 2024	No data concerning CBD clearance

Supplementary Figure 1: RoB 2.0 for Risk of Bias evaluation among included Randomized Controlled Trials

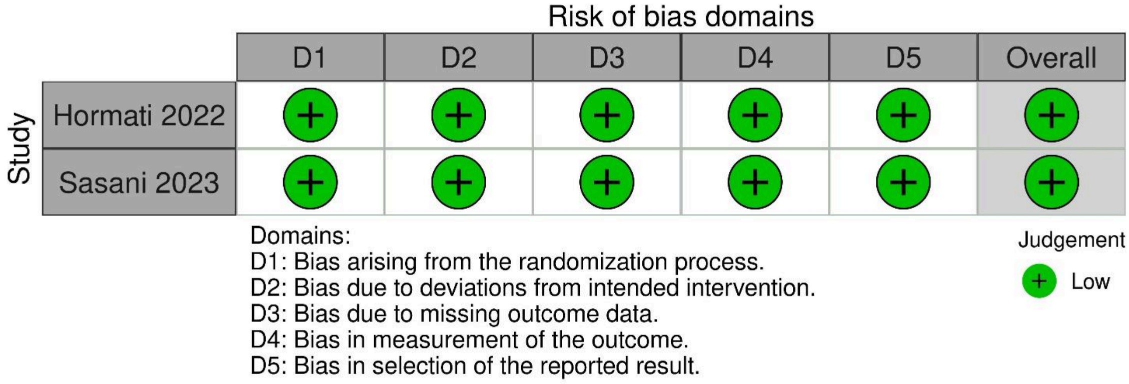

Supplementary Figure 2: ROBIN for risk of Bias evaluation among included NRSI

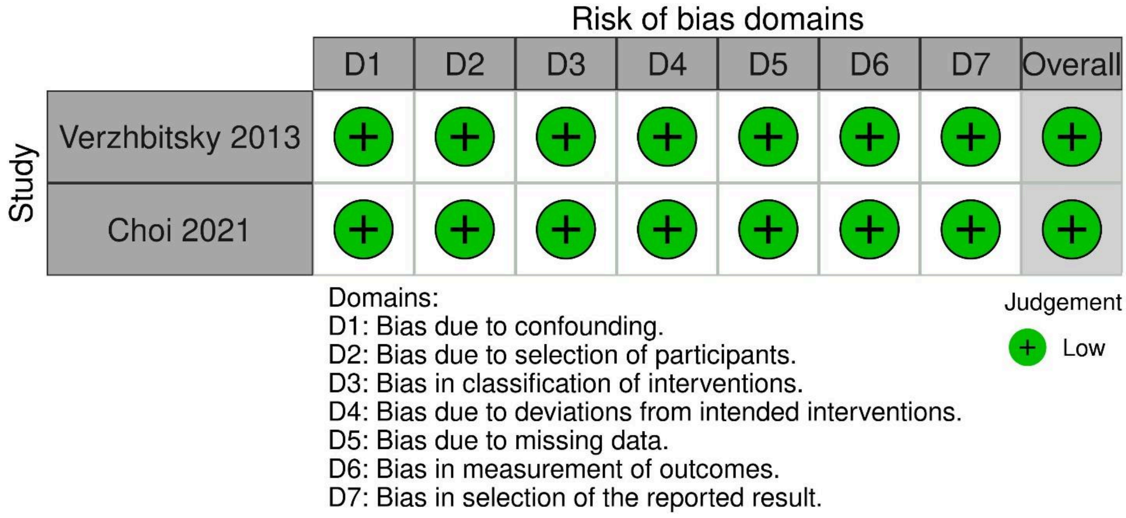

Supplementary Figure 3: Clinical recurrence in studies including pigtail as intervention group

	Double pigtail stent		No Stent			Odds Ratio
Study or Subgroup	Events	Total	Events	Total	Weight	M-H, Random, 95% CI
Choi 2021	20	264	34	219	55.9%	0.45 [0.25, 0.80]
Sasani 2023	10	35	6	35	44.1%	1.93 [0.62, 6.07]
**Total (95% CI)**		**299**		**254**	**100.0%**	**0.85 [0.20, 3.55]**
Total events	30		40			

Heterogeneity: Tau² = 0.86; Chi² = 5.00, df = 1 (P = 0.03); I² = 80%
Test for overall effect: Z = 0.22 (P = 0.83)

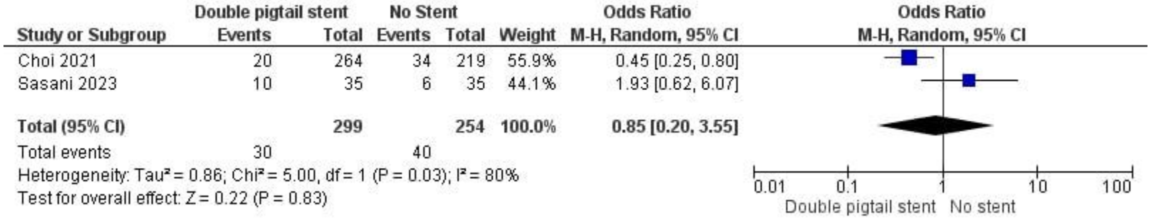

Supplementary Figure 4: Sensitivity analysis after the exclusion of only one study that does not give information concerning residual gallbladder stones

	stent		no stent			Odds Ratio	
Study or Subgroup	Events	Total	Events	Total	Weight	M-H, Random, 95% CI	Year
**1.1.1 RCT**							
Hormati 2022	1	20	6	20	18.2%	0.12 [0.01, 1.14]	2022
Sasani 2023	10	35	6	35	37.2%	1.93 [0.62, 6.07]	2023
**Subtotal (95% CI)**		**55**		**55**	**55.4%**	**0.58 [0.04, 8.74]**	
Total events	11		12				
Heterogeneity: Tau² = 3.09; Chi² = 4.79, df = 1 (P = 0.03); I² = 79%							
Test for overall effect: Z = 0.40 (P = 0.69)							
**1.1.2 NRSI**							
Verzhbitsky 2013	22	110	9	52	44.6%	1.19 [0.51, 2.81]	2013
**Subtotal (95% CI)**		**110**		**52**	**44.6%**	**1.19 [0.51, 2.81]**	
Total events	22		9				
Heterogeneity: Not applicable							
Test for overall effect: Z = 0.41 (P = 0.68)							
**Total (95% CI)**		**165**		**107**	**100.0%**	**0.94 [0.30, 2.95]**	
Total events	33		21				

Heterogeneity: Tau² = 0.57; Chi² = 4.75, df = 2 (P = 0.09); I² = 58%
Test for overall effect: Z = 0.10 (P = 0.92)
Test for subgroup differences: Chi² = 0.25, df = 1 (P = 0.62), I² = 0%

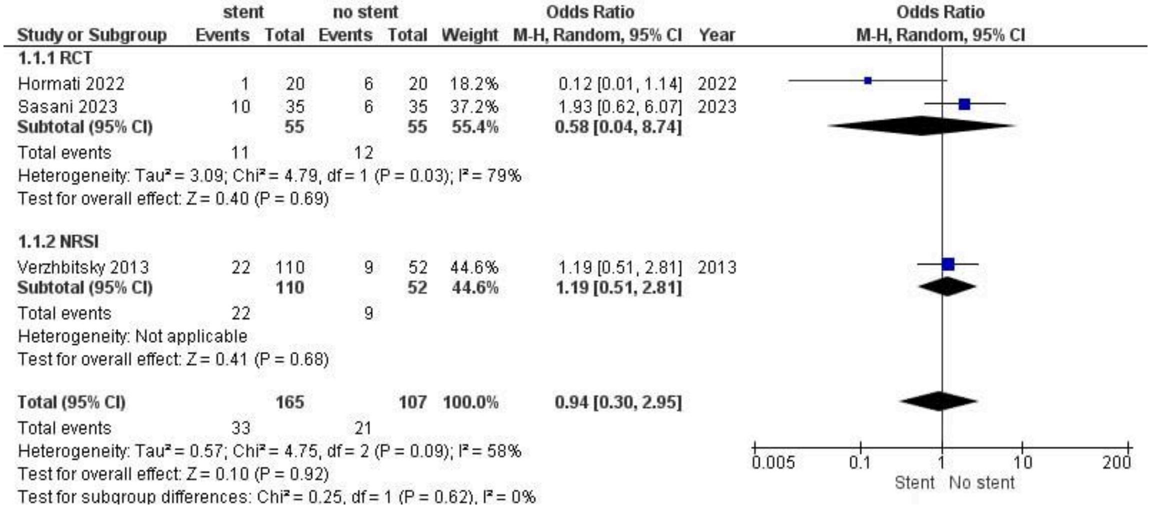

Supplementary Figure 5: Trial sequential analysis

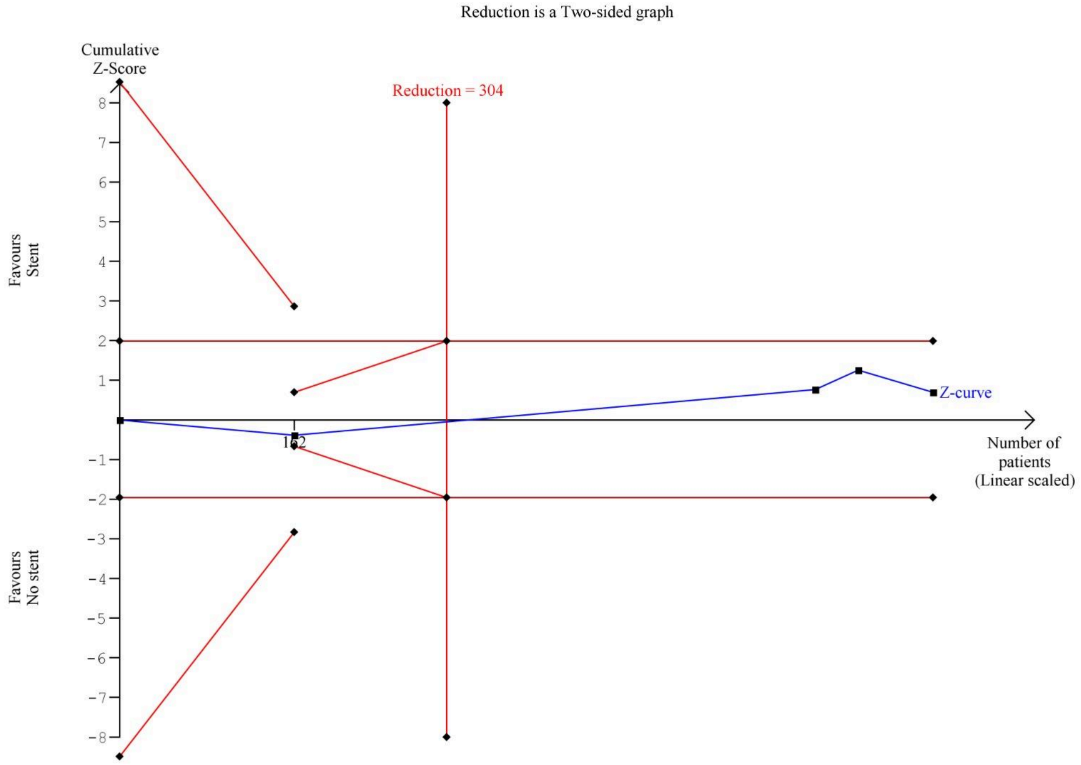

Supplement: Supplementary file 1 — Supplementary Material [file 10-1055-a-2586-6007_25904133.pdf]
